# Hourly Associations between Heat Index and Heat-Related Emergency Medical Service (EMS) Calls in Austin-Travis County, Texas

**DOI:** 10.3390/ijerph20196853

**Published:** 2023-09-28

**Authors:** Kijin Seong, Junfeng Jiao, Akhil Mandalapu

**Affiliations:** 1 Urban Information Lab, School of Architecture, The University of Texas at Austin, Austin, TX 78712, USA; jjiao@austin.utexas.edu; 2 Department of Public Health, The University of Texas at Austin, Austin, TX 78712, USA; akhilm@utexas.edu

**Keywords:** distributed lag non-linear model, heat index, intensity, hourly excess heat, extreme heat, emergency medical service (EMS) incident

## Abstract

This paper aims to investigate the following research questions: (1) what are the hourly patterns of heat index and heat-related emergency medical service (EMS) incidents during summertime?; and (2) how do the lagged effects of heat intensity and hourly excess heat (HEH) vary by heat-related symptoms? Using the hourly weather and heat-related EMS call data in Austin-Travis County, Texas, this paper reveals the relationship between heat index patterns on an hourly basis and heat-related health issues and evaluates the immediate health effects of extreme heat events by utilizing a distributed lag non-linear model (DLNM). Delving into the heat index intensity and HEH, our findings suggest that higher heat intensity has immediate, short-term lagged effects on all causes of heat-related EMS incidents, including in cardiovascular, respiratory, neurological, and non-severe cases, while its relative risk (RR) varies by time. HEH also shows a short-term cumulative lagged effect within 5 h in all-cause, cardiovascular, and non-severe symptoms, while there are no statistically significant RRs found for respiratory and neurological cases in the short term. Our findings could be a reference for policymakers when devoting resources, developing extreme heat warning standards, and optimizing local EMS services, providing data-driven evidence for the effective deployment of ambulances.

## 1. Introduction

In recent years, health challenges imposed by anthropogenic climate change have garnered increased public attention and have become an emerging global public health problem [1]. With an increasing concern for heat-induced illnesses due to climate change, the literature provides evidence that there are both immediate and lagged effects of heat on human health. Some studies have noted that extreme heat has an immediate effect on all-cause and cardiovascular morbidity [2]. The results reported by Cui et al. [2] are corroborated by other sources in the literature and support the notion that extreme heat is associated with cumulative detrimental effects on cardiovascular morbidity [3,4].

Several studies have examined how extreme heat affects different health outcomes, particularly focusing on cardiovascular and cardiorespiratory symptoms, investigating severe health outcomes related to extreme heat or exploring the association between temperature and cause-specific mortality using various approaches [5,6]. Additionally, other studies have looked into the impact of heat on various health outcomes, including hospitalizations, all-cause mortality, and neonatal outcomes [7,8,9]. These studies have observed a slightly higher risk when exposed to higher temperatures in relatively short lag periods with different associations depending on the outcome variable, thus lending support to the investigation of how the association between extreme heat and hospitalizations changes across critical categories of adverse health outcomes. 

Regarding hospitalizations, emergency medical services (EMS) are typically the first responders to any health emergency and often the first point of contact with an individual and the healthcare system during an unforeseen adverse health outcome. In the case of heat-related incidents, EMS can typically provide care to immediately cool down an individual and stabilize the patient until they can arrive at a hospital or medical center [10]. Given that, the EMS data can provide information about an individual’s health status and treatment options before death [11]. The timeliness of EMS data makes them a vital indicator for assessing downstream health impacts resulting from extreme heat [11]. These factors highlight the need to predict demand during extreme heat events. Throughout the literature, there is support for a positive association between temperature and demand for ambulance services [12]. Research indicated that a 10 °C increase in temperature was associated with nearly a 5% increase in ambulance calls [13]. Moreover, a nearly 9% increase in emergency vehicle dispatching can be found for every one °C above a certain threshold temperature [14]. Nevertheless, coupled with staffing shortages in emergency medical services in cities, the increasing demand for services places a tremendous strain on EMS resources during extreme heat events. In addition, existing studies have highlighted the inadequacies of EMS systems in the United States compared to in other countries. This disparity primarily arises from the fragmented nature of the U.S. EMS system, where varying protocols, equipment standards, and training regimens are prevalent at the state or municipal level [15,16]. For EMS, the United States relies on a combination of public and private funding, while some countries have centralized systems subsidized by government-funded healthcare. Thus, it is crucial to understand how the current EMS system in the U.S. responds to heat-related patients during extreme heat events. 

Our review of the literature revealed the existence of several gaps in the current body of knowledge. First, although the influence of heat index patterns on health has been recognized, the specific effects of heat index features such as frequency, intensity, and duration on heat-related symptoms have not been thoroughly investigated. Second, the potential of hourly weather forecasts in enhancing EMS response remains untapped, with studies generally focusing on daily time series data, which may overlook short-term effects of heat exposure. Finally, while geographic variations in heat exposure and health relationships are acknowledged, there is a dearth of specific study in areas such as central Texas where extreme heat is becoming an escalating concern. These gaps highlight the need for comprehensive studies on the specific impacts of heat index and the value of hourly weather data and region-specific investigations for improvement of our understanding and mitigation strategies for heat-related health issues.

Thus, this study advances the inquiry into the temporal patterns of heat indices in the summertime and the hourly lagged effect of heat indices on heat-related health illnesses. This paper aims to address the gap in the literature by investigating the following research questions: (1) what are the hourly patterns of the heat index and heat-related EMS incidents during summertime? and (2) how do the lagged effects of heat intensity and hourly excess heat vary by heat-related symptoms?

## 2. Materials and Methods

This study investigates Austin-Travis County, Texas, where the city of Austin is located, as the area has seen tremendous economic and population growth in recent years. Given that, the area has experienced challenges associated with rapid urban growth, including urban sprawl and the increasing demand for public services. 

The timeframe of this study is limited to the period from May to September in 2020 and 2021. This timeframe was selected as it has historically been the typical hot season for Austin-Travis County, Texas. It thus is when extreme heat events are most likely to occur.

### 2.1. Data on Hourly Emergency Medical Service Calls

Emergency medical services (EMS) incident data were obtained from Austin-Travis County Emergency Medical Services (ATCEMS), the primary EMS provider for Travis County. The incident dataset was preprocessed using Python. The dataset was filtered to only include incidents between May and September, inclusive, in 2020 and 2021. Referring to a historical monthly weather report spanning from 1981 to 2019, previous research indicates that the hot season in Texas typically extends for around five months, from May to September [17,18]. The heat-relevant health problems were coded with the following medical issues: ‘Abdominal Pain’, ‘Allergic Reaction’, ‘Altered Mentation’, ‘Attended Patient’, ‘Burn’, ‘Cardiac Arrest’, ‘Chest Pain’, ‘Diabetic’, ‘Environmental Exposure’, ‘Headache’, ‘Heart Problems’, ‘Respiratory’, ‘Seizure’, ‘Sick’, ‘Special Event Medical’, ‘Stroke’, ‘Syncopal Episode’, ‘Unconscious’ (n = 49,514). These medical issues were selected based on the literature as proxies for extreme heat exposure since extreme heat-related illnesses often manifest as other illnesses and, consequently, are underreported [19]. The existing literature presents evidence supporting both immediate and delayed effects of heat on human health. Cui et al. (2020) [2] reported an immediate impact of extreme heat on overall morbidity and cardiovascular health, and these findings are supported by corroborative evidence from other studies emphasizing the harmful cumulative effects of extreme heat on cardiovascular morbidity [3,4,20]. In addition, exposure to high temperatures for extended periods of time is a causal factor for a variety of heat-related illnesses, including heat cramps, heat stress, heat syncope, heat exhaustion, dehydration, heat stroke, and death [19,21,22,23]. Previous research conducted on exertional heat stroke also has highlighted its severeness; it causes central nervous system (CNS) dysfunction (e.g., altered level of consciousness, disorientation, confusion, hysteria, irritability, aggressiveness, and seizure) [24,25,26,27]. Given that, incidents were also subdivided into the following categories based on the International Classification of Diseases, 11th revision (ICD 11): cardiovascular (n = 12,093), respiratory (n = 7587), neurological (n = 4619), and non-severe (n = 20,019) cases [28]. All other incidents (n = 5196) were included in the all-cause analysis as these incidents were non-specific but may be related to extreme heat exposure.

### 2.2. Data on Meteorological Factors and Heat Index

Weather data were collected from the U.S. Climate Reference Network (USCRN) at a sub-hourly interval (5 min) for the study’s timeframe. The weather data were collected at a weather station located in the northwest of Travis County, Texas (latitude: 30.629, longitude: −98.081). This station was selected as Travis County only has one weather station for USCRN data, and this station was the closest in proximity. There were 31 observations with missing air temperature data (from seven days: 25 June 2020, 1 July 2020, 7 July 2021, 20 July 2021, 26 July 2021, 5 September 2021, and 23 September 2021). Linear interpolation between observations surrounding missing observations was used to address this limitation. As an alternative temperature indicator, the heat index was used to examine the effects of heat-related illness. Originally developed in 1978 and later adapted by the U.S. National Weather Service (NWS), the heat index is utilized as an indicator for climate conditions to analyze the combined effect of air temperature and relative humidity on human wellbeing [29]. Since humidity significantly contributes to the body’s ability to cool down through evaporation and perspiration, it is necessary to consider the interaction between temperature and humidity, especially when extreme heat events occur [30]. The heat index was calculated by utilizing the maximum air temperature and relative humidity (Equation (1)) and the heat index formula developed by the U.S. NWS and was implemented in the Python package “meteocalc” (ver. 1.10):

HI = −42.379 + (2.04901523 ∗ T) + (10.14333127 ∗ R) − (0.22475541 ∗ T ∗ R) − (6.83783 ∗ 10^−3^ ∗ T^2^) − (5.481717 ∗ 10^−2^ ∗ R^2^) + (1.22874 ∗ 10^−3^ ∗ T^2^ ∗ R) + (8.5282 ∗ 10^−4^ ∗ T ∗ R^2^) − (1.99 ∗ 10^−6^ ∗ T^2^ ∗ R^2^) (1)

where HI indicates the heat index, T is the maximum air temperature in Fahrenheit, and R is relative humidity [31]. 

The sub-hourly dataset was transformed into an hourly dataset by utilizing two key measures: intensity and hourly excess heat (HEH). Heat index intensity was calculated by selecting the maximum heat index among the five-minute timescale values within an hour. That is, for each hour, the highest heat index at the sub-hourly level was designated as the intensity measure for that hour. 

HEH was calculated at a five-minute interval by subtracting the threshold value of 100.0 °F (37.8 °C) from the heat index. This value is the threshold for issuing a heat advisory based on the NWS’s general heat advisory guidance [32,33].

This study followed the methodology outlined by Tang et al. for calculating daily excess hourly heat (DEHH), however, at an hourly timescale [1]. If the heat index was below the threshold value, the excess heat value was recorded as 0.0. For each hour, the overall cumulative excess hourly heat was calculated by adding together consecutive sub-hourly excess heat values greater than 0. If there was a period in which the heat index was below the threshold, the cumulative count was reset. The highest of the cumulative excess heat values was designated as the overall cumulative hourly excess heat (Equation (2)).

(2) $$\mathrm{HEH}=\sum_{0}^{55}∆h_{i}\;{∆h}_{i}=\left\{\begin{array}{cc} h_{i}-h_{t}, & h_{i}\;\geq h_{t}\\ 0, & h_{i}<h_{t} \end{array}\right.$$

where *i* refers to the min. time of observation (5-min basis); *h_i_* is the heat index at the observation time; *h_t_* is the heat threshold, which is 100.0 °F (37.8 °C); and △*h_i_* is the difference between *h_i_* and *h_t_*.

HEH was used to provide additional insights beyond the heat index. While the heat index accounts for the combined effects of temperature and humidity, excess heat measures capture how much heat over the threshold is accumulated over time. Despite its complexity, the HEH allows us to capture the additional heat stress beyond a certain threshold and its potential impact on heat-related incidents, providing a nuanced understanding of the relationship between excessive heat exposure and heat-related incidents, particularly in the context of public emergency preparedness.

In addition, several contextual data points were added to the hourly dataset. First, the number of heat-related EMS incidents, as well as the number of incidents in each of the subcategories (cardiovascular, respiratory, neurological, unspecified) within each hour, was recorded. Secondly, information regarding the day of the week, public holidays, and year was added to control for potential confounding values. Day-of-week values ranged from 0 to 6, with Monday being 0 and Sunday being 6. Public holidays in the United States were indicated using a binary variable, with 1 indicating a public holiday such as Memorial Day (25 May 2020 and 31 May 2021), Independence Day (4 July 2020 and 2021), and Labor Day (7 September 2020 and 6 September 2021).

### 2.3. Statistical Analysis

This paper utilized a distributed lag non-linear model (DLNM) to examine the statistical relationship between hourly heat index patterns and heat-related EMS incidents, which can describe exposure–response dependency throughout lag space as well as lag–response dependence among heat index exposures [34]. Lagged associations between excess heat and intensity were explored using the distributed lag non-linear model (DLNM) using the ‘dlnm’ package (ver. 2.4.7) developed by Gasparrini in R (ver. 4.2.1) [35]. 

Several models were tested with various configurations; the model with the lowest quasi-Akaike’s Information Criterion (Q-AIC), indicating better model fit, was selected [1,2,20]. The model that was selected controlled for the year, month, day of the week, and public holidays. A natural spline for the year with one degree of freedom and a natural spline for the month with three degrees of freedom were utilized, along with categorical variables for the day of the week and a binary variable for the public holidays. The DLNM for this study was constructed as follows (Equations (3) and (4)):

*Log[E(y_t_)]* = α + *β*_1_*CB*(INTENSITY_t,l_) + *β*_2_*NS*(year, 1) + *β*_3_*NS*(month, 2) + *β*_4_DOW + *β*_5_PH (3)

*Log[E(y_t_)]* = α + *β*_1_
*CB*(HEH_t,l_) + *β*_2_*NS*(year, 1) + *β*_3_*NS*(month, 2) + *β*_4_DOW + *β*_5_PH (4)

where *t* indicates the calendar day of observation, *E(y)* is the predicted counts of heat-related EMS incidents at hour *t*, α is the intercept of the model, *β*_1_*–β*_5_ are the coefficients of the statistical regression model, INTENSITY and HEH are the cross-basis (*CB*) matrix in DLNM, l represents the lag days, and *NS* means the natural cubic spline function in the model. In this study, the calendar year and month were used as fixed time strata and control periods comprising the day of the week (DOW) and public holidays (PH) as categorical variables.

While weather conditions are independent of the day of the week, the day of the week and public holidays were accounted for due to different behavioral patterns that depend on the day of the week. During weekdays, many individuals in the working-age population spend time in air-conditioned workplaces or schools whereas on the weekend they may spend more time outdoors. Additionally, healthcare providers and facilities may have limited capacity on weekends, resulting in delays in treating particular conditions which may predispose individuals to utilize EMS during extreme heat events.

After structuring the model, the overall cumulative and single lag associations between heat index patterns and heat-related EMS incidents from all-cause, cardiovascular, respiratory, neurological, and non-severe causes were plotted and estimated. 

Model evaluation was performed regarding the low (5th percentile) and high (95th percentile) values of both heat intensity and HEH, with reference to the 50th percentile (median) and the interquartile range (IQR) values, respectively.

### 2.4. Data on Meteorological Factors and Heat Index

We ran a sensitivity analysis to adjust the year and month in the models. We changed the degree of freedom (df) for the year (as we only had two years, 2020 and 2021, in our study, the df for the year was automatically set to 1) and month (2, 3, 4, 5). Based on the lowest quasi-Poisson Akaike information criteria (QAIC), the optimal df of 3 for a month and the maximum lag of 72 h were chosen to capture the effect of heat index on heat-related EMS incidents. All sensitivity analyses were carried out in R ver. 4.2.2 with the “dlnm” package.

## 3. Results

Table 1 summarizes the hourly meteorological factors and heat-related EMS cases in Austin, Texas, during 2020 and 2021. The hourly mean temperature, relative humidity, heat index intensity, and HEH of the summer season were 78.38 °F (25.77 °C), 64.10%, 80.73 °F (27.07 °C), and 53.78 °F (12.1 °C), respectively. The average hourly value of heat intensity and interquartile range (IQR) of duration were 79 °F (26.11 °C) and 103.36 °F-minutes (39.64 °C), respectively. The hourly minimum and maximum heat index intensities were 51 °F (10.56 °C) and 105.62 °F (40.9 °C), respectively, during the whole study period. The range between the minimum and maximum HEH varied from 0 to 302.78 °F-minutes (−17.78 to 150.43 °C-minutes).

There were 49,514 cases of all-cause EMS calls in the summertime from May to September. The hourly mean count of heat-related EMS calls was 6.74. The hourly average number of non-serious EMS calls was 2.73, showing the highest number among the four specific causes of heat-related EMS calls, and cardiovascular cases occurred at a rate of about 1.65 cases per an hour. The maximum count of heat-related EMS calls was 24 cases in an hour.

**Figure 1:**
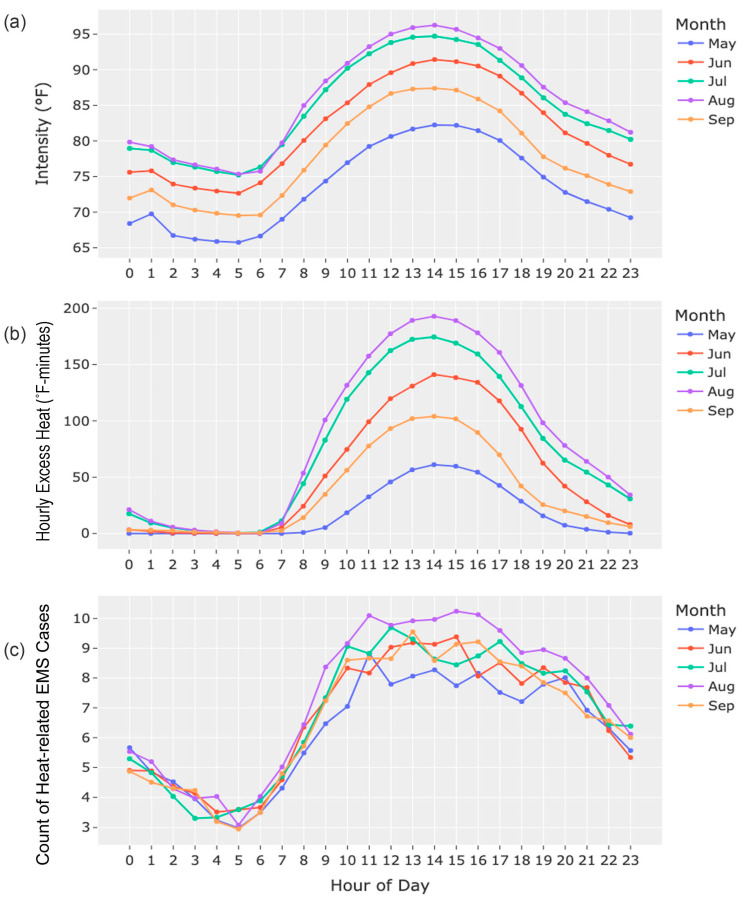
The average hourly heat index intensity (**a**), the average hourly excess heat (**b**), and the average hourly counts of heat-related EMS cases for all causes (**c**), in five summertime months (May, June, July, August, and September) in Austin, Texas, during 2020 and 2021.

Figure 1 shows the hourly average of heat index intensity, HEH, and heat-related EMS incidents for all causes during the summertime of 2020 and 2021 in Austin, Texas. The average heat index intensity peaked at 1 to 4 PM in the five months for all groups, exceeding 95 °F (35 °C) in August. Regarding the daily patterns in heat index duration (see Figure 1b), daily heat was not accumulated until 7 AM, but the hottest heat index duration peaked at 1 to 3 PM in the five months, showing the highest accumulation of heat of approximately 200 °F-minutes (93.33 °C-minutes) at 2 PM in a day.

In addition, the heat index’s intensity and duration followed the same monthly trends, with August being the hottest month, followed by July, June, September, and May. Heat-related EMS incidents (see Figure 1c) showed similar patterns to the heat index intensity and HEH given that the average cases increased during the daytime. However, their monthly trends were not consistent hourly, with the highest number of events occurring during the day in August.

**Figure 2:**
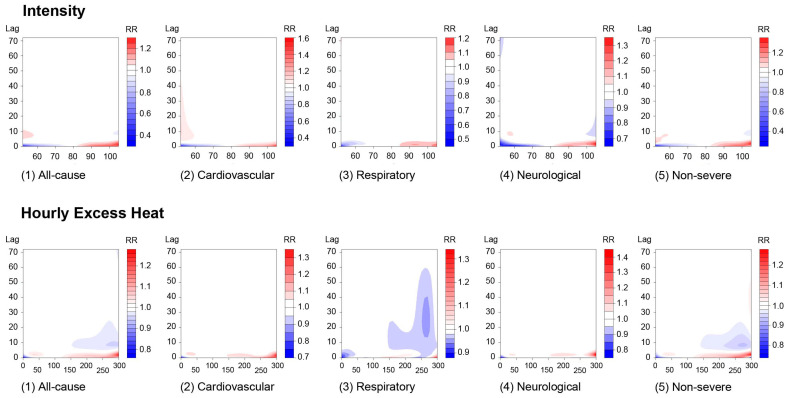
The contour graphs of the exposure–response relationship between heat index patterns (intensity and duration) and five types of heat-related EMS incidents with references at 79 °F (26.11 °C) and 103 °F (39.44 °C), respectively, in Austin, Texas.

The contour graphs of the exposure–response relationship between heat index patterns and five types of heat-related EMS incidents are presented in Figure 2. Overall, in both heat intensity and HEH, there are positive associations between heat index patterns and heat-related EMS incidents at relatively small lag periods, ranging between lag 0 and 10 h, showing higher relative risks (RRs) for higher heat index intensity and duration. Generally, slightly elevated risks of EMS incidents are observed primarily to increase as the intensity exceeds 80 °F (26.67 °C) and HEH exceeds 150 °F-minutes (65.56 °C-minutes) in each case. Regarding the lagged effects in both intensity and duration, these elevated risks seem to be restricted to a lag of under 10 h.

**Figure 3:**
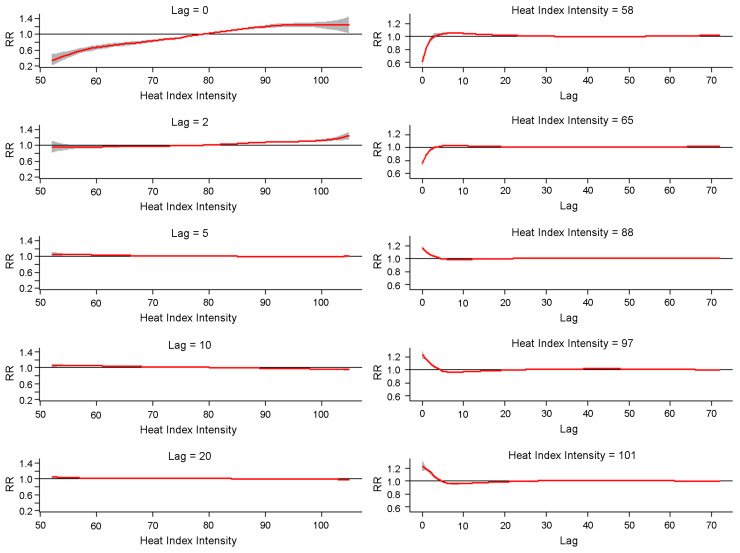
Lag-specific and heat-index-intensity-specific relationship for all-cause EMS incidents in Austin, Texas. Red lines stand for the estimated effects. Grey areas are 95% confidence intervals.

For all-cause and cause-specific EMS incidents, our results follow the general trend, with a slightly higher RR associated with under 10 h lag at higher intensities. The effect of heat index intensity at lag 0 h shows a negative association with low intensity (<79 °F (26.11 °C)) for all-cause heat-related EMS incidents (see Figure 3). When the intensity reaches 101 °F (38.33 °C), there is an immediate risk of heat-related illness at a very short-term exposure (<5 h); then, the RR is not higher than 1.0 after that (see Figure 3). Higher heat index intensity can increase the risk of heat-related illnesses, among which the impact of a higher heat index appears immediately for every symptom, including in all-cause cases, while there is a notable, later-appearing risk with low intensity at a lag ranging from roughly 5 to 40 h for cardiovascular EMS incidents.

Given that heat index duration indicates a cumulative heat index generated by adding excess heat within an hour, the trends shown in the result can be explained by the way HEH is calculated. Similar to the result of intensity, high HEH correlates with higher RR at very short lags but a relatively lower RR at much larger lags in 10 to 20 h. This finding is replicated mainly in the respiratory and non-serious heat-related symptom categories.

Table 2 shows the cumulative effects of heat index intensity and duration at different lag periods on hourly heat-related EMS incidents. Generally, higher heat intensity causes immediate, short-term lagged effects on all causes of heat-related EMS incidents, whereas lower heat intensity has protective effects in all case subgroups. 

For all-cause heat-related symptoms, low intensity (5th percentile = 65 °F, 18.33 °C) has protective effects during lag 0–48 h (RR was 0.82 (95% CI: 0.73, 0.94)). In addition, all-cause, cardiovascular, respiratory, and non-severe cases show a higher RR during the lag 0–10 h (RR: 1.34 (95% CI: 1.24, 1.45), 1.24 (95% CI: 1.07, 1.43), 1.24 (95% CI: 1.03, 1.50), and 1.50 (95% CI: 1.33, 1.68), respectively), while neurological cases show more immediate detrimental impacts within 5 h (RR: 1.47, 95% CI: 1.28, 1.69). In all cases and subgroups, the highest RR for high heat intensity is shown at the lag 0–5 h. 

As for HEH, the protective effects with lower risks (5th percentile = 0 °F, −17.78 °C) at the lower HEH last during the lag 0–72 h for all-cause, respiratory, and non-severe cases (RR was 0.70 (95% CI: 0.59, 0.82), 0.48 (95% CI: 0.32, 0.70), and 0.58 (95% CI: 0.45, 0.74), respectively). Meanwhile, cardiovascular and neurological cases have a relatively shorter period of significantly lower risks at the lag 0–10 h (RR was 0.79 (95% CI: 0.69, 0.91) and 0.71 (95% CI: 0.57, 0.88), respectively). 

At higher duration (95th percentile = 199 °F-minutes, 92.78 °C-minutes), there are immediate adverse effects at lag 0–5 h in all-cause, cardiovascular, and non-severe cases, with statistically significant high RRs of 1.20 (95% CI: 1.12, 1.28), 1.24 (95% CI: 1.09, 1.41), and 1.18 (95% CI: 1.07, 1.31), respectively. Respiratory and neurological cases have no statistically significant RR within 72 h, except at the lag 0–48 h. 

## 4. Discussion

This study explored how heat index patterns, including intensity and duration, cause lagged effects on heat-related symptoms based on local EMS data. A non-linear hourly association between heat index patterns and heat-related EMS incidents was found, controlling for year, month, day of week, and public holiday effects. Our results also showed hourly variations and a lagged relationship between higher heat index and heat-related EMS incidents, consistent with previous studies that tested in different areas or timescales [1,2,6,20]. This is the first study to examine the hourly association between heat index patterns and the main heat-related symptoms using EMS cases in the Southern United States.

A novel approach of measuring cumulative heat exposure using hourly excess heat (HEH) builds upon prior methods of DEHH by measuring heat on a finer timescale. This method accounts for cumulative heat exposure, which may contribute to chronic heat stress. This method may become increasingly critical during heat waves that persist for much longer periods of time. As such, this metric may inform EMS of potential unexpected high-risk situations. For instance, it may occur when the current heat index may not be acutely high, but EMS demand may be high due to temperatures not cooling down sufficiently, preventing individuals from recovering to prior heat exposures. Likewise, this metric can be used to design EMS responses during extreme heat waves to be prepared for short, acute heat exposure over a day and long, persistent heat exposure over several days.

Furthermore, we have underscored the significance of applying the HEH method at an hourly level to enhance services effectively. By providing hourly resolution, HEH offers a more detailed temporal perspective, enabling us to gain deeper insights into heat-related phenomena with higher granularity. We emphasize how this enhanced temporal resolution is a key asset in improving services across multiple domains. For instance, it facilitates real-time decision-making for emergency responders, allows for more timely and targeted public health interventions, and enhances our ability to allocate resources efficiently. In essence, the hourly HEH methodology plays a pivotal role in addressing the dynamic nature of heat-related incidents and significantly contributes to the overall enhancement of services in the context of heat-related risks and impacts.

Our study found that higher heat index intensity and HEH are associated with elevated relative risks (RR) for heat-related EMS incidents, showing positive correlations with the lag time of heat-related EMS incidents at relatively shorter lag periods. Additionally, there were several notable trends specific to particular call categories. Exposure to heat may contribute to or exacerbate cardiovascular disorders by causing dehydration, electrolyte loss, and increased surface blood circulation [36]. While corroborating the previous findings, the slight increase found at lower intensities in categories such as cardiovascular symptoms in our study might potentially be explained by physiological changes due to cold temperature or other uncontrolled factors. Generally, colder temperatures are associated with an increased risk of cardiovascular mortality due to changes in blood viscosity and vasoconstriction [37]. The colder temperatures in our model are generally much warmer than the temperatures associated with cold risk in other studies. However, this increased risk could be due to the cold temperatures being outliers relative to the warm temperatures typically found in the summertime in Austin, Texas. Moreover, due to the nature of our dataset focusing on summertime, our result could be limited in estimating the effect of the adverse effects of colder temperatures compared to the model’s performance in relation to warmer temperatures.

In terms of the lagged effects, our findings suggest that as temperature increases beyond a certain threshold, there is an associated risk of adverse health outcomes that increases as the intensity continues to increase, consistent with the findings from previous studies [1,2,6,20]. Similarly, with HEH, the elevated risk appears to begin after experiencing a large amount of cumulative excess heat and rapidly increases as cumulative excess heat increases. Many studies demonstrated that hot temperatures have short-term effects on mortality and morbidity, whereas cold temperatures have delayed and long-lasting adverse effects with high RR [38,39,40]. In addition, the low temperature in most previous studies was considered extremely cold temperature (approx. 27 °F/−2.78 °C) during an entire year, which increases RR more, showing “U-shaped” RR patterns [2,8]. Compared to this, a couple of studies used summer only as a study period and had similar patterns to our results that show a positive association between temperature and the occurrence of heat-related diseases, especially in terms of short lag effects [40]. Nevertheless, the fact that our study only focused on summertime temperatures could be a limitation, as we could not estimate the impact of lower temperatures in wintertime. The lower temperatures observed during the summer season fall within the normal temperature range and may differ from the lower temperatures experienced during wintertime in other studies. Thus, it is crucial to consider this difference when comparing our findings to research examining the effects of low temperatures during the wintertime.

The results of the cumulative effects in higher HEH show that the lag range of the higher RR varies by symptom categories. Notably, neurological incidents have relatively higher RR in longer periods at the lag 0–48 h (RR = 1.98; 95% CI: 1.05, 3.74), while other symptoms show an immediate adverse effect within 5 h. Not much research has investigated temperature-induced neurological symptoms (seizures) or whether excessive heat causes seizures to occur in people with epilepsy. However, we assume that heat causes dehydration, which may raise the likelihood of a seizure. When bodily fluid loss (mainly sweat) exceeds fluid intake, sodium and glucose levels decline [41]. It eventually leads to low blood sugar levels (hypoglycemia), which may produce seizures in certain persons, triggering neurological biotransformation later [41]. 

Our findings suggest that during extreme heat events, EMS demand will increase in a relatively short amount of time. The slightly increased association with EMS demand across all disease categories indicates that extreme heat has the potential to place an immense burden on EMS and healthcare systems in the short term [12,13]. There also appears to be a certain threshold beyond which there is an increased risk both in terms of intensity and duration [14]. As such, EMS departments and public health officials can use these findings to anticipate surges in demand to better improve EMS responsiveness and, ultimately, patient outcomes. Given that, our findings suggest that EMS providers prepare for periods of high heat by ensuring adequate staffing (i.e., adjusting work shifts or bringing in additional staff), equipment, and medical supplies to handle the surge in demand. Moreover, collaborating with weather forecasting agencies can help EMS systems to anticipate periods of increased demand and adjust their resource allocation accordingly. In addition, it might be beneficial to revise the EMS protocols to ensure a fast and efficient response during extreme heat events, developing a heat-related illness triage protocol that can quickly identify and prioritize high-risk patients. Moreover, given that socially vulnerable populations are at a higher risk of heat-related illnesses, efforts should be made to ensure that these areas have adequate access to EMS. By evaluating EMS availability and response times across different regions, additional resources (such as ambulances and staff) could be allocated to areas with a higher proportion of vulnerable populations to improve EMS response times. 

Urban planners and policymakers should provide timely EMS for all. In regard to emergency departments in hospitals or emergency stations, it is recommended that the prioritization and allocation of medical resources and the advancement of medical services should be established robustly. Given that, it is important to take into account the temporal patterns of heat-related illnesses in consideration of year, seasonality, day of the week, and holidays [42,43]. Additionally, geographic patterns of EMS incidents should be considered for the equitable provision of medical services. Seong et al. (2022) [17] found that social vulnerability factors, such as race (i.e., Hispanic and Black), social benefit status, and living alone, are highly associated with heat-related EMS incidents, causing geographical patterns of inequality in heat-related health Socially vulnerable populations often reside in underserved living environments with no ventilation, cooling equipment, or nutritional supplements with more vitamins and liquids. They are subjected to extreme heat that necessitates immediate medical intervention to prevent the adverse effect of high temperature and humidity [29]. Moreover, as discussed by [3], when the temperature reaches a certain threshold, a “harvesting effect” is seen as heat might promote early morbidity onset in vulnerable groups, resulting in RR < 1 following short-term exposure [3]. Given that heat-vulnerable areas are clustered by region [17], geographical disparities in heat-related illnesses and EMS resources should be mitigated through future studies that consider the regional stratification of vulnerable populations during extreme heat events. One of the suggestions to improve immediate EMS response is to adapt data-driven prediction for EMS demands using advanced datasets and data analytics. For instance, using real-time, high-resolution weather forecasts, such as High-Resolution Rapid Refresh (HRRR), developed by the National Oceanic and Atmospheric Administration (NOAA), may allow researchers to predict extreme heat events in advance more accurately. In addition, recent research employed a more advanced method, an enhanced two-step floating catchment area (E2SFCA), to assess EMS accessibility while taking EMS station and hospital service capacity into account [44]. Hence, future research should aim to employ a comprehensive approach to measuring EMS accessibility to better prepare immediate medical EMS response for climate resilience.

Limitations exist in this study. First, although Austin-Travis County is a metropolitan area with a fairly large population of 964 K, the hourly number of ambulance cases was, on average, six for all-cause incidents in our study period. Such a small sample size might lead to sampling bias with higher variability; specifically, it could occur in specific-case subgroups with smaller sample sizes. However, this was redeemed by conducting sensitivity analysis, iteration of the model selection process, and comparison of the results with daily-level analysis [2]. Second, heat-related cases may not be fully captured by the count of EMS incidents since patients who use private and public transportation cannot be included in this study. In particular, given that EMS use varies by socioeconomic status due to language barriers or financial constraints [17], future research should consider stratified samples when using EMS cases as proxies of heat-related symptoms. Third, due to the lack of detailed spatial information in the study, exposure misclassification may have occurred by uncontrolled factors, such as wind direction, wind speed, barometric pressure, or air pollutants [6]. Future studies should seek to use a rich dataset, including temporal and spatial control factors, for better prediction. Finally, despite the careful selection of heat-relevant health problems (Table A1), we recognize that our study is inherently limited by the nature of heat-related illnesses and the lack of full diagnosis information. Multiple factors can contribute to a healthy outcome, and it may not be clear whether every call included in the study was directly due to extreme heat exposure. Thus, it should not be used for direct clinical interpretation but rather as a guide to inform public health interventions and preventive measures against heat-related illnesses.

## 5. Conclusions

This paper adds to the existing knowledge on the lagged effects of high temperatures on heat-related illnesses. Delving into the heat index patterns in terms of intensity and hourly excess heat, our findings suggest that higher heat intensity has immediate, short-term lagged effects on all causes of heat-related EMS incidents, including cardiovascular, respiratory, neurological, and non-severe symptoms, while its RR vary by time. In addition, HEH also shows a short-term cumulative lagged effect within 5 h in all-cause, cardiovascular, and non-severe symptoms, while the RR for respiratory and neurological cases are not statistically significant, except at the lag 0–48 h for neurological cases. This study corroborates previous studies on associations between temperature and heat-related health outcomes and broadens the conversation to include EMS cases and hourly scale lagged effect.

Our study is unique in that it first investigated the effects of hourly excess heat, accumulated in 5 min intervals using the heat index, on heat-related illnesses. Such a detailed analysis of the temporal changes in heat distinguishes this research from previous studies. Second, our study’s novel contribution lies in its examination of these associations within the context of EMS incidents. By explicitly focusing on EMS calls related to cardiovascular, respiratory, neurological, and non-severe causes, we provide valuable insights into the impact of temperature on emergency medical services by category. Third, our methodology allowed us to analyze lagged effects and cumulative excess heat, providing a more comprehensive understanding of the relationship between temperature and heat-related EMS incidents over time. This temporal perspective enhanced our ability to identify patterns and potential thresholds at which the risk of adverse health outcomes increases.

Overall, our study adds to the existing literature by providing detailed insights into the specific context of heat-related EMS incidents. The methodology employed allowed for a nuanced analysis of temperature effects and contributes to a better understanding of the implications for EMS and potential strategies for improvements in response and preparedness.

The research findings have significant implications for improving the current EMS system. By identifying the relationship between temperature and heat-related EMS incidents, we can enhance preparedness by providing valuable insights into seasonal patterns and increased risks during periods of high heat. This information can help EMS agencies and healthcare providers better allocate resources and ensure they are adequately staffed and equipped to handle potential surges in demand. Additionally, understanding the specific health outcomes associated with heat exposure allows for targeted interventions, including tailored protocols, improved triaging processes, and appropriate stockpiling of equipment and medication. Furthermore, our findings also contribute to public education and awareness campaigns on heat safety, foster collaborative partnerships between EMS agencies, healthcare providers, and community organizations, and support continuous quality improvement efforts within EMS systems.

In sum, this study investigated the vital role of heat intensity and hourly excess heat on heat-related health outcomes using EMS cases. Our findings can be a reference for policymakers and practitioners when devoting resources, developing extreme heat warning standards by symptoms, and optimizing local EMS by providing data-driven evidence for the effective deployment of ambulances. Our findings from the hourly scale study shed light on a better-designed EMS response during extreme heat events for long-term community resilience and equitable public health services.

## Figures and Tables

**Table 1:** Statistical summary of hourly meteorological factors and EMS calls in Austin, TX, during 2020 and 2021.

Variables	Mean	SD	Percentiles				
			Min.	P25	P50	P75	Max.
Meteorological Factors							
Temperature (°F)	78.382	8.889	52.160	72.680	77.540	84.740	105.620
Relative Humidity (%)	64.098	22.048	7.000	47.000	66.000	84.000	97.000
Heat-Index Intensity (°F)	80.728	9.836	51.185	74.247	79.291	88.387	105.146
Hourly Excess Heat (HEH) (°F-minutes)	53.779	71.543	0.000	0.000	3.511	103.362	302.779
Ambulance Calls (per hour)							
All-Cause Cases (N = 49,514)	6.743	3.781	0	4	6	9	24
Cardiovascular Cases (N = 12,093)	1.647	1.427	0	1	1	2	10
Respiratory Cases (N = 7587)	1.033	1.121	0	0	1	2	8
Neurological Cases (N = 4619)	0.629	0.831	0	0	0	1	6
Non-Severe Cases (N = 20,019)	2.726	2.054	0	1	2	4	14

**Table 2:** Cumulative effects of heat index intensity and duration at different lag periods on hourly heat-related EMS calls by symptom in Austin, TX, during 2020 and 2021.

Heat Index Pattern	Lag Hours	RR * (95% CI **)				
		All-Cause	Cardiovascular	Respiratory	Neurological	Non-Severe
Intensity (5th percentile = 65)	0–1	**0.67 (0.63, 0.71)**	**0.64 (0.57, 0.72)**	0.95 (0.82, 1.09)	**0.63 (0.52, 0.75)**	**0.70 (0.63, 0.76)**
	0–5	**0.67 (0.63, 0.70)**	**0.66 (0.59, 0.73)**	**0.83 (0.73, 0.95)**	**0.61 (0.52, 0.72)**	**0.67 (0.61, 0.72)**
	0–10	**0.75 (0.70, 0.81)**	**0.74 (0.65, 0.84)**	**0.85 (0.73, 1.00)**	**0.65 (0.53, 0.79)**	**0.77 (0.70, 0.86)**
	0–24	**0.87 (0.79, 0.96)**	0.89 (0.74, 1.08)	0.87 (0.68, 1.10)	**0.72 (0.53, 0.97)**	0.91 (0.78, 1.05)
	0–48	**0.82 (0.73, 0.94)**	0.97 (0.77, 1.23)	0.83 (0.61, 1.13)	0.79 (0.54, 1.15)	**0.80 (0.66, 0.97)**
	0–72	0.95 (0.82, 1.11)	1.07 (0.81, 1.42)	1.01 (0.70, 1.44)	1.01 (0.65, 1.56)	0.94 (0.75, 1.17)
Intensity (95th percentile = 97)	0–1	**1.43 (1.36, 1.50)**	**1.43 (1.30, 1.58)**	**1.17 (1.04, 1.33)**	**1.21 (1.04, 1.42)**	**1.48 (1.37, 1.59)**
	0–5	**1.62 (1.55, 1.70)**	**1.53 (1.40, 1.67)**	**1.38 (1.23, 1.54)**	**1.47 (1.28, 1.69)**	**1.78 (1.66, 1.90)**
	0–10	**1.34 (1.24, 1.45)**	**1.24 (1.07, 1.43)**	**1.24 (1.03, 1.50)**	1.18 (0.93, 1.49)	**1.50 (1.33, 1.68)**
	0–24	1.03 (0.91, 1.17)	0.99 (0.79, 1.25)	0.99 (0.74, 1.32)	0.98 (0.68, 1.41)	1.13 (0.94, 1.36)
	0–48	**1.17 (1.01, 1.35)**	1.29 (0.98, 1.70)	0.92 (0.65, 1.31)	1.42 (0.92, 2.21)	1.15 (0.92, 1.43)
	0–72	**1.18 (1.01, 1.38)**	1.24 (0.92, 1.66)	1.16 (0.79, 1.68)	1.15 (0.71, 1.86)	1.16 (0.92, 1.47)
Hourly excess heat (5th percentile = 0)	0–1	**0.68 (0.65, 0.70)**	**0.65 (0.61, 0.70)**	**0.80 (0.74, 0.87)**	**0.65 (0.59, 0.72)**	**0.69 (0.65, 0.72)**
	0–5	**0.62 (0.60, 0.65)**	**0.68 (0.62, 0.74)**	**0.67 (0.60, 0.74)**	**0.61 (0.54, 0.70)**	**0.58 (0.55, 0.62)**
	0–10	**0.69 (0.64, 0.74)**	**0.79 (0.69, 0.91)**	**0.65 (0.54, 0.77)**	**0.71 (0.57, 0.88)**	**0.62 (0.56, 0.69)**
	0–24	**0.78 (0.69, 0.87)**	0.95 (0.77, 1.18)	**0.59 (0.46, 0.77)**	1.00 (0.71, 1.40)	**0.66 (0.56, 0.78)**
	0–48	**0.71 (0.62, 0.82)**	0.83 (0.64, 1.08)	**0.51 (0.37, 0.71)**	1.32 (0.88, 1.99)	**0.60 (0.48, 0.74)**
	0–72	**0.70 (0.59, 0.82)**	0.86 (0.64, 1.17)	**0.48 (0.32, 0.70)**	1.39 (0.86, 2.25)	**0.58 (0.45, 0.74)**
Hourly excess heat (95th percentile = 199)	0–1	**1.11 (1.06, 1.17)**	**1.09 (1.00, 1.20)**	1.03 (0.92, 1.17)	1.02 (0.88, 1.18)	**1.11 (1.03, 1.19)**
	0–5	**1.20 (1.12, 1.28)**	**1.24 (1.09, 1.41)**	1.05 (0.89, 1.24)	1.18 (0.96, 1.45)	**1.18 (1.07, 1.31)**
	0–10	1.04 (0.93, 1.16)	1.12 (0.91, 1.38)	0.92 (0.71, 1.19)	1.06 (0.76, 1.46)	0.99 (0.85, 1.17)
	0–24	0.88 (0.74, 1.05)	1.02 (0.73, 1.41)	0.70 (0.47, 1.04)	1.13 (0.67, 1.90)	0.79 (0.61, 1.02)
	0–48	0.98 (0.79, 1.21)	1.14 (0.77, 1.70)	0.61 (0.37, 1.01)	**1.98 (1.05, 3.74)**	0.85 (0.62, 1.16)
	0–72	0.87 (0.69, 1.10)	1.02 (0.66, 1.58)	0.69 (0.40, 1.19)	1.50 (0.74, 3.02)	0.75 (0.53, 1.06)

* RR: relative risk; ** 95% CI: 95% confidence interval. Bold means statistically significant (*p* < 0.05).

## Data Availability

The data presented in this study are available on request from the corresponding author.

## Appendix A

**Table A1:** Selection of heat-relevant health problems.

Group	Examples of Heat-Related Health Problems	Basis for Inclusion	References
A	Chest Pain, Cardiac Arrest, Syncopal Episode, Stroke, Heart Problems, Respiratory, Abdominal Pain, Diabetic	Chronic diseases susceptible to heat	National Center for Environmental Health (NCEH) [45]; Agency for Toxic Substances and Disease Registry (ATSDR) [46]; Centers for Disease Control and Prevention (CDC) [47]
B	Unconscious, Altered Mentation, Headache, Seizure	Potential symptoms of heat stroke	National Institute for Occupational Safety and Health [48]
C	Environmental Exposure, Burn	Potential symptoms of sunburn	National Center for Environmental Health (NCEH) [45], Agency for Toxic Substances and Disease Registry (ATSDR) [46]
D	Allergic Reaction, Sick, Special Event Medical, Attended Patient	Catch-all for other symptoms related to heat stroke or exhaustion	D’Amato et al., 2015 [49]; Kenney et al., 2014 [50]
